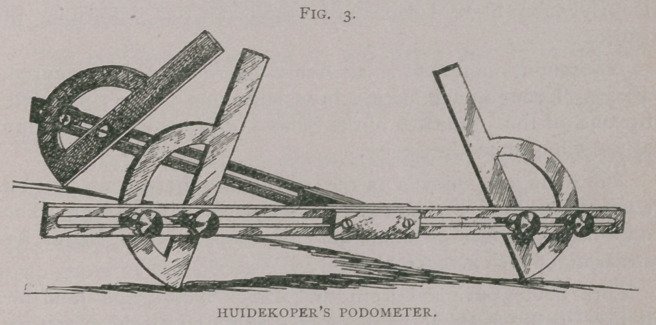# Horseshoeing1A paper read at the April meeting of the New York County Veterinary Medical Association.

**Published:** 1895-05

**Authors:** R. S. Huidekoper

**Affiliations:** Veterinarian


THE JOURNAL

OF

COMPARATIVE MEDICINE AND

VETERINARY ARCHIVES.

Vol. XVI. *MAY*, 1895. No. 5.

### HORSESHOEING.[1]

BY R. S. HUIDEKOPER, M.D., VETERINARIAN.

Horseshoeing dates from the development of the Christian era. The earliest histories we have of the use of horses date back probably 1600 or 1700 before Christ. In the first era of civilization the horse traveled over Europe, reaching the northern part of Denmark; but down to historical times the horse was probably only used irregularly, and, naturally, horseshoeing was not needed.

The first representation we have of a horse in historical times was about 2000 years before Christ. Simultaneously among the monuments of Nineveh and Babylon we have representations of the horse so distinct that they show the true Asiatic race. Following that are representations of the horse in Greek art, in Grecian sculpture. After Greek art had begun to affect Italian art we find representations of the horse so distinct that even the ornaments of the bit, with the reins and caparisons, appear. Yet there is no representation of shoes on a horse up to that time. Afterward, in Xenophon's account, which has been already referred to, and in all the accounts down to the time of Mithridates the Great, where he speaks of the cavalry being stopped on account of the horses' feet being sore, we have no representation whatever of the shoe of the horse. It is not

[1] A paper read at the April meeting of the New York County Veterinary Medical Association.

until about the second century that we find traces of the shoeing of the horse in certain shoes which are now in the Museum at Mayence, Germany, which evidently were only used as a protection to feet which had become sore—two shoes, one of them a flat plate with a metal cap coming to the front part of the hoof, and sets of rings to fasten it to the pastern; the other, a shoe consisting of a cup with four sets of rings to fasten it to the pastern. Such shoes undoubtedly could not have been used at all for any purpose beyond that of protecting a sore foot. About the fifth century, in Rome, we find horseshoes which began to approximate to those used to-day. The traces of horseshoes which have been found show evidently that they developed among the Huns, in the northern part of France among the Normans, in the neighborhood of the fifth century. A few centuries later, about the tenth century, at about the time of the early Crusades, from 1091 to 1200, the horseshoe had been developed to a far greater state of perfection, and the use of calks came in. The shoes of the first century were held on with two nails, sometimes with four nails. In the tenth, eleventh, and twelfth centuries shoes were found with ten and sometimes with six nails, with calks at the heels and toes. At an earlier period shoes were found among the Huns during their migrations, and they were in general use among the Normans, and in the northern part of Spain and in Italy. In the neighborhood of the fifteenth century the shoe had reached almost the perfection of the ordinary horseshoe of to-day. Among the Crusaders from Italy we find a horseshoe with four nails, evenly made. Since that time there has been little change except in certain modifications in special parts of the shoe.

I think I can best illustrate to you what the theory of balancing or levelling the foot is, by showing the foot in its transverse section, looking at it directly in front.

I give a representation of the foot with a few blocks of wood (Figs. 1 and 2). Fig. 1 represents a level foot as it stands on the ground, and Fig. 2 an uneven one. Of course, that outside part of the hoof, the wall, contains the pedal bone, which is the essential part, and that—Mr. Bonner was one of the first to use the word "suspension"—is suspended in the foot by the laminæ which holds it to the wall of the foot and is not supported by the bottom of the foot. The bone is not kept from going down, because there is a bit of sole underneath it, but it is kept from going down because it is suspended by a series of laminæ

which, if spread out, would cover a space of ten or twelve square feet. Then, fitting on top of that, we have the bone which we represent by the second block (Fig. I B), the pastern bone; then there is the second bone (Fig. I A) the os suffraginis. Then the shank bone, or the cannon bone, has on either side of it two small bones, the splint bones, which are only attached by fibrous tissue, and on the top of these is the knee.

FIG. I.

FIG. 2.

I am going above the foot, because, while I acknowledge all the importance given to the foot by the gentlemen who have spoken before me, there is a great deal more in a horse besides his foot. The stomach of a horse is of just about as much value as anything else. A horse with chronic lameness of the foot who feeds well three times a day will go forty miles as easily as a sound horse that does not eat well. There are a great many diseases, as has already been said, which originate above the foot, and which produce diseases of the foot secondarily. In a horse that has been lame for some time it is often a very difficult question to determine which has been the primary cause and which has been the secondary effect. Disease of the foot, in a majority of cases, produces trouble above. But in many cases the disease, which has originated above, will cause alterations in the foot below. If the foot is perfectly level treat the leg as you would a piece of machinery. But the piece of machinery must play perfectly and evenly on a perfectly even bearing. Treat the trouble exactly as they treated the piston which ran the propeller of the *Gascogne*, which recently came into port so far behind schedule time, and which I see experts attribute to its bearings having been inaccurate. The instant you alter those bearings, and any slackness occurs on one side or the other, you will have an extra wear and tear. The horse swings his legs freely. He has a foot that comes down on its proper surface perfectly level. He takes a firm hold and goes on, and his leg swings out again just exactly as a bit of machinery or as the piston of a steam engine will work. The instant the bearings are altered, or it is made a little crooked, we alter the bearings of every joint from above. Suppose the foot to be represented by the block of wood (Fig. 2). Instead of a foot which is perfectly level (Fig. 1), suppose we take a foot which has been allowed by the blacksmith to become crooked, so that it stands higher on one side than on the other (Fig. 2 *a*, *c*). Then the whole leg above is thrown out of bearing. What is the consequence? The instant a horse comes down on his foot he strikes with the highest part of the foot (Fig. 1 A). That acts as a fulcrum. The foot bears over and we have two troubles as a consequence. In the first place, the leg tries to keep straight and it presses over to the side of the lower heel (Fig. 2 *c*). We have a pressure of the bone here into the foot. The bone, resting as it does on the pedal bone, instead of coming down squarely and playing there in its joint, is thrown down sideways, and there is a pressure on the pastern or navicular bones, and there may be a bruising of the bones. If the weight happens to strike a joint higher we have bruising the bone at the pastern, producing ringbone, or, instead of bone bruising, we have stretching of the ligaments on the opposite side. The periosteum of the bone is torn. We will go a bit higher than that. At the upper part of the leg we have a central shaft, the cannon bone, and we have two small splint bones at the side held by a fibrous tissue, and on top of that is placed the knee-joint. By a crooked foot the leg is thrown over. The leg is thrown down on the top of these splint bones, and we have a splint thrown out, because the horse fails to come down properly. If any of you have had gout, if you have had a sore toe, and you have to go along on one side of the foot or back on your heel, you know perfectly well how quickly you get sore up in your knees or your hips, and you do not know in what part of the leg the extra soreness lies. Yet all of that is secondary soreness coming from walking on a sore toe. Just so in the case of the horse; the instant there is soreness in one part the animal attempts to save himself, and there comes a pain at some other point. Within a day or two I have had a horse with a lame foot whose shoulder on the opposite side was so sore that for a few days he could hardly bear the touch of a finger. He was working the muscles of one leg in order to save himself on the other side. There is much more to be said on this point, but I shall not occupy any more of your time in regard to it, but will now go to the solution of it, which I suppose is what you want to know.

The solution is that you must get farriers who are better educated. Now, I do not believe in all the statements about the farrier being such a beast as he is ascribed to be, or about his being absolutely vicious. There is just as much viciousness and ignorance in the majority of horse-owners as there is in the farrier. The horse-owner should take an interest in the shoeing, and should know that it is properly done.

I have had a great deal of experience with farriers, and I have found some very good ones. I have found better men in the country than in the city. Out in the country you get a farrier who not only shoes the horses in his neighborhood, but also does a bit of wheelwrighting and odd jobs of tinkering of wagons, and uses iron in various ways, training his son or a neighbor's son as an apprentice. He teaches these boys to be mechanics, and when they go to work to shoe a horse they are good workmen; they have some intelligence. But the labor-unions in the shops in our large towns and cities try to prevent the teaching of apprentices. They teach a boy to-day how to shoe when he cannot make a proper shoe. We ought to have our farriers properly educated. This is one of the few countries where the farriers are not properly educated. In Germany, Italy, France, and Russia they have schools in which farriers are taught. They teach them the anatomy of the horse's foot, so that when they nail a piece of iron on the foot they know what they are doing. They teach them the principles of the mechanical movement of the leg above, so that they are able to appreciate the deviations and the springing in and out of the leg, which indicates what may be the trouble with the foot. In the veterinary schools of France we had to go to the black-smith's shop and work two hours every morning. We had to learn how to forge a shoe properly, and to make horse nails, and when we got our diplomas we had to know something about shoeing. In the schools of London, Glasgow, Edinburgh and the schools in America they teach theoretically. In the school where I studied, as I said, we had to learn how to forge the shoes, and then to put them on a horse. In Philadelphia I made my students go to the blacksmith shop and make their own shoes and put them on. Within a few months the blacksmiths of Philadelphia and veterinary practitioners have established a school for farriers.

I think if we were to have a school for farriers we would find a very large number of young men who would take the course, and competent farriers could be turned out. If every man would take a little more interest in his own horses and in their shoeing, instead of leaving it entirely to people who are rather ignorant, and who are not interested in the matter, better shoeing for the horses would be the result. Every man has his own theory of shoeing. To my mind the theory of shoeing should be first to know the normal foot of the horse, which, in size and proportions, varies with the different breeds of horses. For instance, in the Shire of Clydesdale breed a large flat foot, with a very low heel, is a perfectly normal foot, and there contraction in the least possible degree is most easily produced, and is productive of corns and soreness in the navicular bone, while the thoroughbred can have a foot like a mule and travel perfectly sound. There should be an allowance made for the breed of the horse as to what his foot should normally be. We should give the farriers an education which would allow them to recognize what the normal foot should be, and how to make the foot perfectly level from side to side and from heel to toe, based upon the breed of the horse and according to what they judge to be the natural action of the horse, an action which is frequently changed and can be very much improved by shoeing. Then, after you have a perfectly good, normal foot, the principle of shoeing is to put on the simplest piece of metal which will prevent that foot from breaking under the artificial use to which we subject the horse. If the horse can go as he does on the plains, and choose his own movement in going, he can go for months and never wear his foot sore; but where we have the horse put on an artificial pavement, with a shoe on weighing three pounds, as I have seen farriers put on in Paris, and that shoe tears out in a week's work, then we have to adopt artificial means of protection. When we come to any accidental change in the horse's gait, or the special uses to which he is to be adapted, then, of course we have to adopt special shoeing.

In levelling any horse's foot, after using my own eye I always let a horse teach me a certain amount. When I take up a foot and find that it is crooked, that it has a high heel or toe, or is high from side to side, I adjust it as far as my eye can properly do so, because I always do the rasping myself, and then get the blacksmith to put on the shoe as I direct and put in the nails. Then I ask the owner to send the horse out to 155th street and back. Then I let the horse teach me where I have made any mistake. If his foot is perfectly level he will come down squarely every time he steps. If his foot is higher at any one point than it should be he will come down at the highest point first and then lean over. The nails will be slightly worn more at the highest point than at the other side, and I know where I have got to level it more. I have a great many disputes with blacksmiths. The blacksmiths frequently think they know as much as I do, and probably more. I had an instrument made to show the blacksmith how to level the foot. (Fig. 3.) It is a

FIG. 3.

HUIDEKOPER'S PODOMETER.

triangle, set off in degrees, which I can apply to the bare foot and take the angles exactly. At the time I had my own farrier in Philadelphia he kept a regular record-book as to every horse that was shod. I had taken the absolute measurement of every horse's foot and had it entered in a large invoice-book, and in three weeks, when that horse came back to be shod again, and had been eased at his heels, so as to allow play, another drawing was made. It was interesting, sometimes marvellous, to see the change in the horse's foot and the expansion that had been effected by a perfectly simple shoe. We kept that up for some two years, and found it to be an excessively interesting study. I found it did a great deal of good with some blacksmiths when they found I could really prove to them that I was right in regard to the horse's foot being crooked. This was the only instrument I ever had made, and blacksmiths would often send to borrow it in order to verify some shoeing that they were doing themselves. Now, I think, if the owners will interest themselves a little more in their horses and in the way they are shod they will not find the farrier such an ignorant man as he is often claimed to be, and the horses will be a great deal better shod.

---

The Senate of New York State has unanimously recommended for passage the act exempting veterinarians from jury duty.

The Societies for the Prevention of Cruelty to Animals are waging intense opposition to the bill now pending before the Pennsylvania State Legislature legalizing the dehorning of cattle.

A recent appointment as an Army veterinarian was secured, after a thorough examination in which some fifteen competed, by one of the graduates of the School of Veterinary Medicine at the Ohio State University.

The strongest animals in the world are those that live on a vegetable diet. The lion is ferocious rather than strong. The ox, horse, reindeer, elephant and antelope, all conspicuous for strength, choose a vegetable diet.

Corn blades stripped from the stalks make good food for horses; being free from dust, horses wintered on the same are less likely to develop heaves. Racehorses half a century ago were fed on corn blades in preference to hay.

France has about 3,000,000 horses, and Germany has about 3,500,000. France has eight horses to 100 inhabitants. Germany has only seven and one-half horses to 100 inhabitants. The number of horses used in cities and towns increases every year in a more rapid proportion than the population of the same, largely attributed to the increased facilities offered in the cities for travel.

## Figures and Tables

**Fig. 1. f1:**
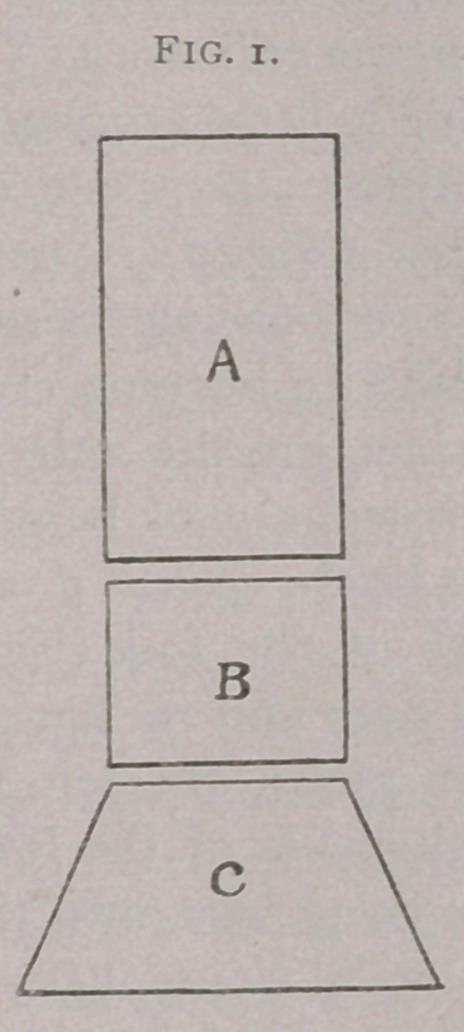


**Fig. 2. f2:**
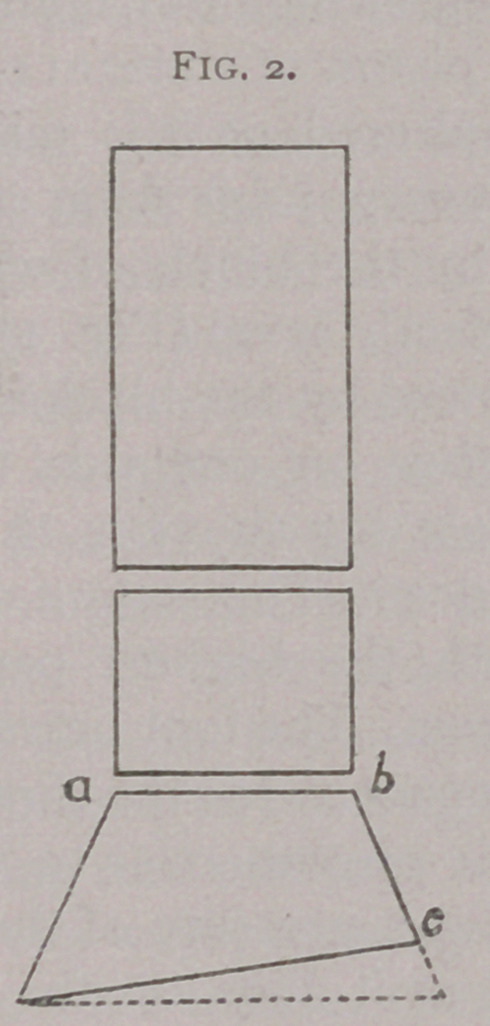


**Fig. 3. f3:**